# Atypical Non-H_2_S-Producing Monophasic *Salmonella* Typhimurium ST3478 Strains from Chicken Meat at Processing Stage Are Adapted to Diverse Stresses

**DOI:** 10.3390/pathogens9090701

**Published:** 2020-08-26

**Authors:** Joana Mourão, Andreia Rebelo, Sofia Ribeiro, Luísa Peixe, Carla Novais, Patrícia Antunes

**Affiliations:** 1 UCIBIO/REQUIMTE, Department of Biological Sciences, Laboratory of Microbiology, Faculty of Pharmacy, University of Porto, 4050-313 Porto, Portugal; jmourao@cnc.uc.pt (J.M.); acr@ess.ipp.pt (A.R.); up201206791@ff.up.pt (S.R.); lpeixe@ff.up.pt (L.P.); casilva@ff.up.pt (C.N.); 2 Center for Innovative Biomedicine and Biotechnology, University of Coimbra, 3004-504 Coimbra, Portugal; 3 Center for Neuroscience and Cell Biology, University of Coimbra, 3004-504 Coimbra, Portugal; 4 Institute for Interdisciplinary Research, University of Coimbra, 3030-789 Coimbra, Portugal; 5 Scientific Area of Environmental Health, School of Health, Polytechnic Institute of Porto, 4200-465 Porto, Portugal; 6 ESCMID Food- and Water-borne Infections Study Group (EFWISG), 4010 Basel, Switzerland; 7 Faculty of Nutrition and Food Sciences, University of Porto, 4150-180 Porto, Portugal

**Keywords:** *Salmonella*, non-H_2_S-producing, ST3478, poultry processing plant, food chain stress, copper, peracetic acid, surveillance, cgMLST and SNPs, comparative genomics

## Abstract

Poultry products are still an important cause of *Salmonella* infections worldwide, with an increasingly reported expansion of less-frequent serotypes or atypical strains that are frequently multidrug-resistant. Nevertheless, the ability of *Salmonella* to survive antimicrobials promoted in the context of antibiotic reducing/replacing and farming rethinking (e.g., organic acids and copper in feed/biocides) has been scarcely explored. We investigated *Salmonella* occurrence (conventional and molecular assays) among chicken meat at the processing stage (*n* = 53 batches/29 farms) and characterized their tolerance to diverse stress factors (antibiotics, copper, acid pH, and peracetic acid). Whole-genome sequencing was used to assess adaptive features and to perform comparative analysis. We found a low *Salmonella* occurrence (4%) and identified *S*. Enteritidis/ST11 plus atypical non-H_2_S-producing *S*. 1,4,[5],12:i:-/ST3478. The ST3478 presented the ability to grow under diverse stresses (antibiotics, copper, and acid-pH). Comparative genomics among ST3478 isolates showed similar antibiotic/metal resistance gene repertoires and identical nonsense *phsA* thiosulfate reductase mutations (related to H_2_S-negative phenotype), besides their close phylogenetic relationship by cgMLST and SNPs. This study alerts for the ongoing national and international spread of an emerging monophasic *Salmonella* Typhimurium clonal lineage with an enlarged ability to survive to antimicrobials/biocides commonly used in poultry production, being unnoticed by conventional *Salmonella* detection approaches due to an atypical non-H_2_S-producing phenotype.

## 1. Introduction

Non-typhoidal *Salmonella* (NTS) remains one of the most frequent causes of foodborne infections globally and is mainly associated with the consumption of contaminated foods of an animal origin, including eggs and poultry meat [1,2]. Currently, in the European Union (EU), NTS is one of the most well-controlled foodborne pathogens due to the implementation of diverse food safety regulations and specific monitoring programs at poultry production (from the farm to the consumption level) [3,4,5]. *Salmonella* control programs implemented at EU poultry production in the last few decades has led to an initial long-term decrease in human salmonellosis (particularly of *Salmonella* Enteritidis). However, according to the last annual zoonosis report of the European Food Safety Authority (EFSA), there was observed a stabilization trend during the years 2014–2018 in the overall incidence of salmonellosis considering all reported cases of the participating countries [2]. At the same time, expansion of less-frequent serotypes, including newly emerging strains with epidemic potential or with atypical biochemical features (e.g., hydrogen sulfide-H_2_S negative), and frequently multidrug-resistant (MDR) strains, have been reported in poultry production over diverse geographical regions [1,2,6,7,8].

Since the control of zoonotic pathogens should be focused on reducing contamination in the entire poultry meat chain, it is essential to investigate NTS occurrence and the factors contributing to their survival, particularly those related to production management systems (farm and slaughterhouse characteristics, including biosafety control measures at the farm or slaughter/processing stage), that are still scarcely explored [9]. Furthermore, antibiotic restriction policies for the control of antibiotic resistance, including in poultry, call for sustainable biosecurity alternatives (e.g., for disinfection, pest control and as feed additives). A recent and increasingly important alternative to reduce, replace, and refine antibiotics on-farm has been the use of non-antibiotic compounds with antimicrobial activity like metals (e.g., copper-Cu) and organic acids, as feed additives and surface/equipment disinfectants [10,11]. However, the effectiveness/efficacy of these recommended poultry control practices on the elimination of *Salmonella*, particularly of EU-targeted serotypes (*S*. Enteritidis, *S*. Typhimurium and its monophasic variant *S*. 1,4,[5],12:i:-), has been scarcely explored [9,11]. Therefore, it is essential to monitor poultry meat contamination rates by NTS, currently performed through ISO standard cultural methods, as well as to characterize the adaptive features contributing to their survival in poultry production.

In this study, we investigated the occurrence of clinically relevant *Salmonella* serotypes among fresh chicken meat samples at the poultry processing stage in Portugal and their ability to tolerate diverse stress factors. Moreover, we report for the first time the presence of an atypical non-H_2_S-producing clinically relevant *S*. 1,4,[5],12:i:- ST3478 strain adapted to diverse poultry production-related stresses, which by comparative genomic analysis was revealed to be an ongoing emerging clonal lineage.

## 2. Materials and Methods

### 2.1. Sampling Strategy in the Slaughterhouse and Processing Plant

Raw chicken meat samples (*n* = 53), recovered after slaughter and chilled, were collected over six months during 2018 (including in spring and summer) in a Portuguese poultry-production slaughterhouse, immediately before distribution for retail sale. The broilers were from 29 intensive-based farms settled in the north and the center of Portugal, with a similar conventional indoor and floor-raised production system (broiler flocks ranged from 2500 to 8000 per house with age at slaughter from 28 to 42 days). Concerning biosecurity measures, peracetic acid with hydrogen peroxide (between 0.5–3%) was used at the processing plant as a biocide for disinfection as well as copper and organic acids (unknown composition) as additives in the poultry feed. Each sample was processed as a pool of neck skin from 10 carcasses of the same batch (each batch corresponded to one flock from the same house and farm). All samples were collected in sterile plastic bags, transported at 4 °C, and processed on the same day at the laboratory. Subsequent sample processing was performed by cultural and molecular approaches as described in the next sections.

### 2.2. Detection and Characterization of Salmonella by a Cultural Approach

**Figure 1:**
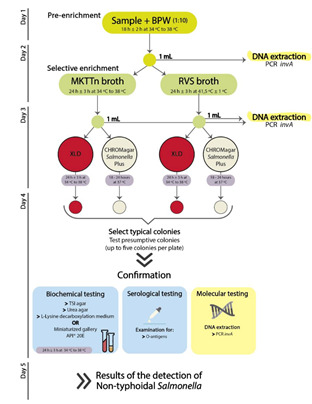
Workflow for the detection and confirmation of *Salmonella* isolates using the ISO 6579-1:2017 standard cultural method and the additional molecular approach.

*Salmonella* detection and characterization was performed using the International Standard Organization-ISO 6579-1:2017 standard method [12]. Briefly, 25 g of sample was initially pre-enriched in Buffered Peptone Water (BPW) (Liofilchem, Roseto degli Abruzzi, Italy) and incubated at 34–38 °C. After that, we performed a selective enrichment by adding 0.1 mL and 1 mL of the previous BPW to Rappaport-Vassiliadis medium with Soya (RVS, 41.5 °C ± 1 °C) and Muller-Kauffmann tetrathionate-novobiocin (MKTTn, 34–38 °C) broths (Biogerm, Maia, Portugal), respectively. The previous selective broths were finally streak-plated on Xylose Lysine Deoxycholate (XLD) (Liofilchem, Roseto degli Abruzzi, Italy) agar and CHROMagar^™^ *Salmonella* Plus (Biogerm, Maia, Portugal). Presumptive *Salmonella* colonies recovered from both selective media (up to five colonies per plate) were confirmed by biochemical tests (e.g., API-20 E—bioMérieux, Marcy l’Etoile, France) as well as agglutination with *Salmonella* O poly antisera and serogroup-specific antisera (BD Difco™, Franklin Lakes, NJ, USA) (Figure 1). *Salmonella* isolates were also additionally confirmed through a molecular approach using a standard PCR for the detection of *invA* marker gene (Figure 1) [13], using the primers and conditions described in Table S1.

### 2.3. Detection of Salmonella by a Molecular Approach

*Salmonella* detection in raw poultry samples was also performed by a molecular approach using a standard PCR for amplification of the *invA* gene, applied directly to DNA extracted from the pre-enriched and enriched broths (BPW and RVS/MKTTn) (Figure 1, Table S1). This DNA extraction was conducted by a boiling-based protocol optimized in this study for poultry high-fat samples. Briefly, we first added 1 mL of BPW, RVS and MKTTn to Eppendorf tubes. After that, we centrifuged the previous suspensions (13000 g, 5 min), rejected the supernatant, and washed the pellet with 200 µL of saline. The previous step was repeated, this time washing the pellet with 200 µL of Triton X-100 (1%). The saline cleaning step was performed again to erase any traces of Triton X-100. A new centrifugation (13000 x g for 5 min) and resuspension of the pellet in 100 µL of sterilized ultrapure water was performed. The supernatant containing the total DNA was recovered after a final boiling of the pellet (100 °C for 20 min) and centrifugation (13000 x g for 5 min). The efficiency of the bacterial DNA extraction was always evaluated by a standard PCR targeting the 16S rDNA gene (Table S1) [14].

### 2.4. Phenotypic and Genotypic Characterization of Salmonella Isolates Recovered from Positive Samples

The search of EU-targeted *Salmonella* serotypes (Enteritidis, Typhimurium and 4,[5],12:i:-) [15] and their antibiotic resistance (*bla*_TEM_, *cmlA1*-*catA*-*floR*, *strA*-*strB*-*aadA-aac(3)*-IV-*aphA1*, *sul1*-*sul2*-*sul3*, *tetA*-*tetB*, *dfrA1*-*dfrA12*-*dfrA17*) and metal tolerance (*pcoD*, *silA*, *merA*, *arsB* and *terF*) markers was performed by standard PCRs (Table S1) in all isolates recovered from positive chicken meat samples [16,17,18].

Antibiotic susceptibility profiles of *Salmonella* isolates were determined by disc diffusion for 16 antibiotics (amikacin-30 µg, amoxicillin-10 µg, amoxicillin+clavulanic acid-30 µg, cefotaxime-5 µg, ceftazidime-10 µg, chloramphenicol-30 µg, gentamicin-10 µg, kanamycin-30 µg, meropenem-10 µg, nalidixic acid-30 µg, pefloxacin-5 µg, streptomycin-10 µg, sulfamethoxazole-300 µg, tetracycline-30 µg, tobramycin-10 µg, and trimethoprim-5 µg). For this, we used the European Committee of Antimicrobial Susceptibility Testing-EUCAST [19] guidelines and, when this was not possible, the Clinical and Laboratory Standards Institute-CLSI guidelines [20]. Minimum Inhibitory Concentration (MIC) for colistin was performed by the reference broth microdilution method [21]. When resistance to three or more antibiotics of different families was observed, isolates were characterized as MDR.

MIC to copper sulphate (Sigma-Aldrich-Merck, Taufkirchen, Germany) was determined by the agar dilution method using an A400 multipoint inoculator (Denley, Sussex, UK) in both aerobic and anaerobic atmospheres (GENbox jar with GENbox anaer and an anaerobic indicator; bioMérieux, France) [16,18]. The Mueller-Hinton II agar plates (bioMérieux, Marcy-l’Étoile, France) were supplemented with CuSO_4_ at different concentrations ranging from 0.25–36 mM and adjusted to pH 7.2 (20 h ± 2 h at 37 °C). To assess the isolates’ growth ability in all MIC assays, we inoculated a first and last plate of Mueller-Hinton II agar without CuSO_4_. 

Minimum growth pH, i.e., the lowest pH with visible growth, was assessed by the microdilution method using Mueller-Hinton-II broth (BD BBL™, Franklin Lakes, NJ, USA) adjusted with HCl from 2.0–6.5 (16 h-20 h ± 2 h at 37 °C). Ten microliters of the previous wells without visible growth were then plated in Brain-Heart Infusion Agar (Liofilchem, Roseto degli Abruzzi, Italy; 24 h-48 h ± 2h at 37 °C) to access the minimum survival pH. Determination of MIC to Peracetic Acid-PAA (15% stock solution; Panreac Applichem, Darmstadt, Germany) was performed by an adaptation of ISO 20776-1:2006 microdilution method, using Mueller-Hinton-II broth supplemented with PAA concentrations between 5–90 mg/L (20 h ± 2 h at 37 °C) [22]. The Minimum Bactericidal Concentration (MBC) was then assessed according to NCCLS:1999 using Brain-Heart Infusion Agar (24–48 h at 37 °C) [23]. The previous MIC/MBC assays to PAA were performed without adjusting the pH, the final pH being measured as between 6.0–7.0 for the different concentrations evaluated. Moreover, the MIC/MBC assays to PAA were also performed by adjusting the culture media pH to 4.5, corresponding to the optimum pH for PAA maximum activity [24]. In both types of assays, the pH was always below the pKa of PAA, described as 8.2 [25]. *Salmonella* Typhimurium LT2 and *Enterococcus faecalis* ATCC 29212 were used as controls in all the assays. All the previous assays were performed in duplicate.

### 2.5. Whole-Genome Sequencing (WGS) for Characterization of Salmonella Isolates

One representative isolate from each *Salmonella* serotype and positive sample was chosen for WGS. The Wizard^®^ Genomic DNA purification kit (Promega Corporation, Madison, WI, USA) was used for DNA extraction according to manufacturer’s instructions and the Qubit 3.0 Fluorometer (Invitrogen, Thermo Fisher Scientific, Waltham, MA, USA) for their quantification. DNA sequencing was then accomplished at Eurofins Genomics (https://www.eurofinsgenomics.eu/) using an Illumina^®^ HiSeq (2 × 150bp) technology. The FastQC software v0.11.8 was used to evaluate the quality of the raw reads after sequencing (https://www.bioinformatics.babraham.ac.uk/projects/fastqc/). High-quality raw reads were then *de novo* assembled using SPAdes v3.14.0 [26], and the final quality was assessed by QUAST (http://quast.bioinf.spbau.ru). The assembled draft genomes were annotated for metal tolerance genes using RAST genome annotation server [27] and the Geneious Software v2020.1.2 for manual curation (https://www.geneious.com/). The web-interface tools from the Centre for Genomic Epidemiology (CGE) (http://www.genomicepidemiology.org) were used to assess the content in antibiotic resistance genes (ResFinder and PointFinder) [28], Multilocus Sequence Typing (MLST), and the core genome ST (cgMLSTFinder) [29]. Confirmation of *Salmonella* serotypes was performed with the online tool of the *Salmonella* In Silico Typing Resource (SISTR) [30].

### 2.6. Comparative Genomic Analysis of Salmonella ST3478 Isolates

A comparative genomic analysis using the core-genome MLST (cgMLST) and high-quality single nucleotide polymorphisms (SNPs) was performed between our isolates and fifteen additional *Salmonella* ST3478 genomes queried from Enterobase (https://enterobase.warwick.ac.uk/). The metadata of the included *Salmonella* isolates was retrieved from Enterobase (isolate name, cgST, HC5, country, year, source) and a further search of antibiotic resistance and metal tolerance genes was conducted as previously described in Section 2.5 (Table S2). For cgMLST analysis, we used the *Salmonella* scheme from Enterobase comprising 3002 loci [29] (https://enterobase.warwick.ac.uk/) as well as the Hierarchical Clustering of cgMLST (HierCC) (HC2, the clusters included all strains with links no more than two alleles apart; HC5, the clusters included all strains with links no more than five alleles apart). These strains were used to develop a minimum spanning tree using MSTreeV2, which was edited using the GrapeTree [29,31,32]. Concerning SNP-based analysis, we used the CSI Phylogeny 1.4 pipeline with default parameters (CGE, https://cge.cbs.dtu.dk/services/CSIPhylogeny) to perform a concatenated alignment between the previous 16 genomes plus the complete reference genome of *Salmonella* 1,4,[5],12:i:- SO4698-09 ST34 (accession no. NZ_LN999997.1) and to create a maximum likelihood tree [33]. The interactive Tree Of Life (iTOL) [34] was then used for tree visualization and annotation with relevant metadata. Lastly, to evaluate the conservation and complete transcription of *phs* operon, encoding for thiosulfate reductase, we extracted their complete nucleotide sequence from all *Salmonella* ST3478 genomes. The BLASTn alignment between all sequences and annotation of the *phs* operon were performed using the Geneious Software v2020.1.2 (https://www.geneious.com/).

### 2.7. Nucleotide Sequence Accession Numbers

The raw reads of *Salmonella* 1,4,[5],12:i:- P1-C10 and *Salmonella* Enteritidis P54-C4 were submitted to Enterobase (Uberstrain number SAL_DB7941AA and SAL_DB7942AA, respectively).

## 3. Results and Discussion

### 3.1. Low Salmonella Occurrence among Raw Chicken Meat by Conventional and Molecular Methods

Human salmonellosis cases have been stabilizing in most EU countries since 2014, but with significant increasing trends in Portugal [2]. Absence of *Salmonella* in poultry meat just before distribution for retail sale remains critical, as it is a major source of *Salmonella* infections. *Salmonella* was detected in 4% (*n* = 2 out of 53 batches) of the fresh chicken meat samples studied over six months (spring and summer seasons). This low occurrence of *Salmonella* in raw poultry products agrees with data from other industrialized countries with pathogen reduction programs [2,9]. The presence of *Salmonella* was confirmed in the same samples by the standard cultural method (ISO 6579) and the PCR assay in total DNA obtained from the selective enrichments, showing that molecular detection is a good alternative to laborious and time-consuming conventional approaches [35]. Moreover, molecular-based assays (such as PCR and real-time-PCR) are powerful tools to be used by poultry companies in combination with cultural-based methods as they overcome the lack in the detection of low counts, viable non-cultivable cells or atypical biochemical *Salmonella* profiles [35]. Nevertheless, molecular methods would still benefit from further improvements in terms of sensitivity at the pre-enrichment step [36].

### 3.2. Atypical Non-H_2_S-Producing S. 1,4,[5],12:i:- ST3478 with the Ability to Tolerate Diverse Food Chain Stresses

Among *Salmonella* recovered isolates *(n* = 9), we identified the monophasic variant of *S*. Typhimurium (*S*. 1,4,[5],12:i:-/ST3478) (Spring sample) and *S*. Enteritidis/ST11 (Summer sample), both serotypes currently covered by EU Regulations, including as a food safety microbiological criterion for fresh poultry meat [4,5]. Both serotypes have been reported by EFSA as among the most frequent in causing human infections in the EU in the last few years [2,9] as well as by the Portuguese authorities [37], justifying the relevance of surveillance studies.

In this study, *S*. Enteritidis isolates presented the typical biochemical *Salmonella* profiles, contrasting with all the *S*. 1,4,[5],12:i:- isolates, which were only detected in CHROMagar™ *Salmonella* Plus (purple color) since colonies were shown to be non-hydrogen sulphide (H_2_S)-producers, and thus lacked the typical black color on XLD agar plates (see Section 3.3.1 for further molecular analysis). This atypical *Salmonella* phenotype is especially worrisome since these strains can escape detection (conducting to low *Salmonella* detection rates) on the traditional medium, supporting the utility of the chromogenic media and a further combination with molecular-based methods, as performed here.

The *S*. 1,4,[5],12:i:- poultry isolates presented the typical antibiotic resistance (*bla*_TEM_ + *strA-strB* + *sul2* ± *tetB*) and metal tolerance (*pcoD* + *silA* + *arsB ± merA*) features of the widespread clinically relevant European clone (ST34) [16,17]. Resistance to the tested antibiotics and tolerance to copper (MICs = 32 mM) was restricted to *S*. 1,4,[5],12:i:- isolates and was absent in the *S*. Enteritidis (Table 1), as described previously [16,18]. The frequent use of copper as a feed additive in food-animal production, as occurred in the Portuguese poultry farms studied, alerts for the potential co-selection of MDR clonal lineages, as suggested for *S*. 1,4,[5],12:i:- clones [16]. Acquired resistance to critically important antibiotics like colistin, fluoroquinolones, and extended-spectrum beta-lactams was not observed in any isolate from both serotypes (Table 1).

Additionally, most of our poultry isolates grew and survived at minimal pH = 4.00, which is in line with the lowest pH growth limit reported by most of the studies including *S*. Typhimurium and *S*. Enteritidis strains [38,39,40,41]. An exception was one *S*. 1,4,[5],12:i:- poultry isolate that survived until pH = 3.50. Several studies reported that *Salmonella* might be able to survive those extremely low environmental pH levels (from 2.50 to 4.00) when previously adapted to a mild pH (pH 5.50 to 6.00) and that these acid-responses could be serotype/strain and type of acid-dependent [42,43,44]. Factors contributing to a mild environmental pH can occur at the farm (e.g., organic acids in feeds, animals gastrointestinal tract) or in the processing plant mostly due to the widespread use of acidic surface disinfectants, approved for the food industry (e.g., Peracetic Acid), which can contribute to the emergence of acid-tolerant strains [45].

*Salmonella* behavior to Peracetic Acid-PAA (organic acid used in biocides for surface and equipment disinfection) was identical in isolates from both serotypes, with MICs varying between 60–70 mg/L and MBCs between 70–90mg/L, when the pH of the medium was not adjusted (Table 1). However, large variability in MICs (7–80 mg/L to 500–1760 mg/L) to PAA have been reported between studies [46,47,48,49], as well as bactericidal concentrations (20–80 mg/L to 200–1.000 mg/L) [48,49,50,51], which can be related to the diversity of methodological approaches (e.g., culture medium, incubation temperature, contact time with the compound, preparation of the inoculum) and the few tested serotypes/strains. Concerning the MIC and MBC values when the media was adjusted to pH=4.50, identified as the optimum pH for PAA maximum activity [24], we observed a decreased trend for all the tested isolates (MIC = 20–40 mg/L and MBC = 40–50 mg/L) (Table 1). This decrease can be explained by the occurrence of higher proportions of the undissociated acid at a lower pH (also dependent on its pKa) being more able to diffuse through the cell and reduce the cytoplasmic pH by intracellular dissociation, thus acting more effectively as a antimicrobial [45]. Furthermore, PAA is one of the most oxidizing biocides used in the food industry [52], attacking microorganisms by oxidizing the cell structure, denaturing proteins and enzymes, and increasing cell wall permeability by disrupting sulfhydryl (-SH) and sulphur (S-S) bonds [53]. However, our results by both approaches (with and without an adjusted pH) showed that in-use concentrations of PAA could, in certain conditions, be ineffective against *Salmonella* strains, since MIC/MBC were included in the range of the suggested concentrations for disinfection products applied in the food and feed area (20–3000mg/L for Product-Type PT 4) [54]. Moreover, besides the biocide concentration, other factors should also be considered regarding the use of disinfectants at food processing plants, like the presence of a high load of organic material and the presence of bacterial biofilms, since these factors might contribute to the persistence of sub-inhibitory concentrations and thus co-select the target pathogens or other relevant bacteria [55].

### 3.3. Comparative Genomics Reveals an Ongoing Emergence of a Non-H_2_S-Producing S. 1,4,[5],12:i:-/ST3478 Clonal Lineage

#### 3.3.1. Non-H_2_S-Producing Phenotype Conferred by a Nonsense Mutation in the phsA Thiosulfate Reductase Gene is Increasingly Reported

**Figure 2:**
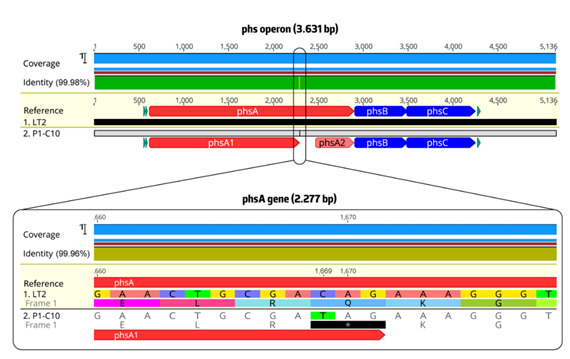
Upper panel—nucleotide alignment and gene synteny of the *phs* operon between *S*. 1,4,[5],12:i:- P1-C10 and the reference strain *S*. Typhimurium LT-2 (accession no. L32188.1). The light blue and green bars represent coverage and sequence identity, respectively. Filled arrows indicate the position and transcriptional direction of open reading frames (red—*phsA* gene, and blue—*phsB* and *phsC* genes). Lower panel—exact location of the nonsense mutation in the *phsA* gene encoding the thiosulfate reductase subunit. The black square with the asterisk represents the stop codon.

All the *S*. 1,4,[5],12:i:- isolates recovered in the present study showed an absence of H_2_S production, which is a rare phenotypic feature among *Salmonella,* regardless of the serotype. However, non-H_2_S-producing *Salmonella* has been reported in the last few years in emerging or outbreak-associated strains recovered from diverse food and human sources worldwide (Table 2) [6,56,57]. Until now, two molecular mechanisms were reported as being responsible for the inability to produce H_2_S in diverse *Salmonella* serotypes (e.g., Aberdeen, Choleraesuis, Infantis, Senftenberg, Typhimurium, and 1,4,[5],12:i:-) and sources (Table 2) [58,59,60]. The most frequent one is associated with mutations in the *phsA* gene—belonging to *phsABC* operon—encoding the precursor of thiosulfate reductase. The other is related to mutations in the *moaC* gene—belonging to *moaABCDE*—affecting the activity of a thiosulfate reductase cofactor. Our non-H_2_S-producing *S*. 1,4,[5],12:i:- isolates, when compared with the H_2_S-producing *S*. Typhimurium LT-2 strain (where this operon was initially described) [61], had a mutation at position 1669 of *phsA* consisting of a single-nucleotide substitution of C to T, resulting in a codon change from CAG (Glutamine-Q) to UAG, a stop codon (Figure 2). This mutation resulted in the premature termination of *phsA* translation; hence the non-H_2_S-producing *S*. 1,4,[5],12:i:- isolates were not able to produce the integral PhsA protein. Of note, the non-H_2_S-producing *S*. 1,4,[5],12:i:- strain from a tomato-associated outbreak in Sweden presented an identical mutation to our strain P1-C10, but other nonsense mutations in the *phsA* gene have also been described in non-H_2_S-producing *Salmonella* from diverse serotypes and sources (Table 2). Since those strains are unable to convert thiosulfate to H_2_S, they might present a potential competitive advantage over other bacteria in the gut. This leverage is probably caused by the increased availability of thiosulfate substrate for *Salmonella* tetrathionate anaerobic respiration (inflammation generates reactive oxygen species leading to the conversion of thiosulfate to tetrathionate, and this is used by *S. enterica* as an electron acceptor in anaerobic respiration), increasing survival ability due to growth and colonization promotion [58,62].

#### 3.3.2. Comparative Genomic of Global S. 1,4,[5],12:i:- ST3478 Showed Common Adaptive Features and Genetic Backgrounds

Our non-H_2_S-producing *S*. 1,4,[5],12:i:- isolates belonged to ST3478 (a Single Locus Variant of the epidemic ST34 from the same eBurst Group eBG1) that is rarely seen in Europe and is only associated with a few sporadic cases so far (*n* = 15; *n* = 8 human clinical cases; *n* = 6 EU countries labs; 2016-2019) (Table S2), as available from Enterobase (http://enterobase.warwick.ac.uk/species/senterica/). Noteworthy, one of those ST3478 isolates was described as an atypical non-H_2_S-producing *S*. 1,4,[5],12:i:- strain recently associated with a large outbreak linked to small tomato consumption produced in an EU country [57], which prompted us to perform further in silico whole-genome analysis. Of note, all fifteen ST3478 genomes available in Enterobase were confirmed by SISTR as *S*. 1,4,[5],12:i:- and presented a *phsA* nonsense mutation at the same position 1669 C > T (Q557X) (Figure 2), suggesting that all are atypical non-H_2_S-producing strains.

**Figure 3:**
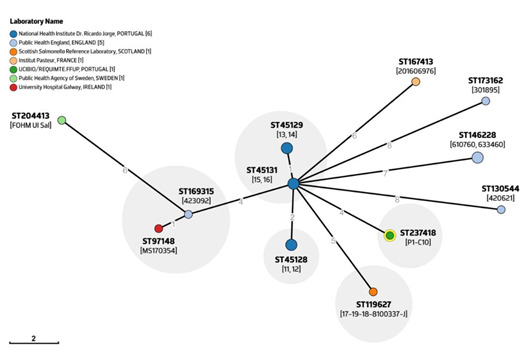
GrapeTree of the *S*. 1,4,[5],12:i:- ST3478 isolates using the cgMLST scheme of Enterobase (isolate names are presented between square brackets). The core genome Minimum Spanning Tree (MST) was created within the Enterobase pipeline using the MSTreeV2 algorithm and GrapeTree tool. The yellow circle corresponds to the cgST237418 of our poultry isolate P1-C10. Clusters of isolates with a maximum of five alleles of distance belonging to the same HC5 group are shaded in grey. For the geographical analysis, the core genome MST was annotated using the lab contact, since country data is missing in several genomes. The scale bar corresponds to the number of cgMLST allelic differences.

The ST3478 genomes were distinguished in twelve cgSTs using the cgMLST scheme from Enterobase (allele-based method), with our poultry isolate P1-C10 belonging to cgST237418 (Figure 3). Interestingly, cgST237418 is more closely related with six isolates of an unknown origin and date of isolation (ID: 11–16) submitted by the Portuguese National Health Institute Dr. Ricardo Jorge (INSA), suggesting that these strains might be already circulating in our country. Moreover, when the Hierarchical Clustering of cgMLST (HierCC) HC2 and HC5 was used, only nine and six HierCC different groups were identified, with the HierCC HC5-45128 group being the most populated, containing ten out of sixteen isolates from Portuguese, Irish, English, and Scottish labs (Table S3). No apparent clustering based on geographical location was observed but, given the low number of ST3478 genomes available in Enterobase, we cannot exclude the possibility that these strains are circulating in other EU countries or continents.

**Figure 4:**
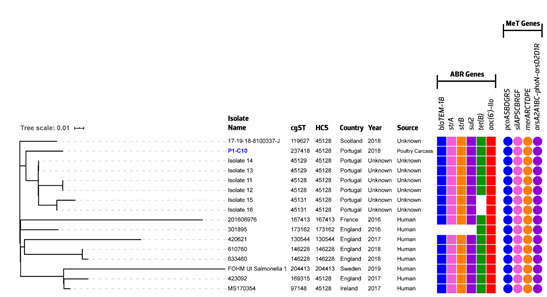
Phylogenetic tree of the *S*. 1,4,[5],12:i:- ST3478 isolates (*n* = 16) based on a SNP-based analysis. The maximum likelihood tree was obtained using the standard pipeline of CSI Phylogeny and *S*. 1,4,[5],12:i:- SO4698-09 ST34 (accession no. NZ_LN999997.1) as the reference genome. Scale bar units represent substitutions per variant site. Associated metadata of all isolates was added using iTOL. Poultry isolate P1-C10 is colored in blue. Each colored filled shape represents the presence of acquired antibiotic resistance (ABR) genes and metal tolerance (MeT) clusters/operons. ABR genes present in only one isolate (*ant(3″)-Ia*) are not represented in the figure. Abbreviations: cgST, core genome Sequence Type; HC, Hierarchical Clustering level by cgMLST.

In order to further confirm the genetic relatedness of ST3478 isolates, we performed an SNP-based analysis. We observed that all ST3478 isolates were closely related, with SNP distances ranging from 0 to 51 (Figure 4, Table S4). Three main clusters were identified as the poultry isolate P1–C10 clustered with ones from unknown sources submitted by INSA-Portugal, with 0 to 14 SNPs differences between the isolates (two + two isolates presented identical core genome SNPs). These data suggest a coherence between clusters formed in cgMLST and SNP-based analysis, as described for other *Salmonella* epidemiological contexts [29,67,68].

Interestingly, all 16 *S*. 1,4,[5],12:i:- ST3478 genomes shared similar genetic repertoires related to antimicrobial resistance, including the integrative and conjugative (ICE) element carrying metal tolerance genes (*pco+sil+ars* clusters) (Figure 4 and Table S2), recently described in *S*. 1,4,[5],12:i:- ST34 [16], besides their close relationship from the phylogenetic (cgMLST and SNPs) point-of-view. When isolates are closely related, frequently, they share parts of their accessory genome, as observed for *S*. 1,4,[5],12:i:- ST34 [69] and other *Salmonella* serotypes [67,68]. The close core and accessory genome might suggest that ST3478 is a newly recently expanded clonal lineage from ST34 with similar antibiotic resistance and metal tolerance features, as is also corroborated by the isolation year of most of the submitted isolates (2016–2019, *n* = 16).

The high occurrence of copper tolerance genes (*pco*+*sil* gene clusters) together with the frequent use of copper as a feed additive in food-animal production alerts for the potential co-selection of ST3478 MDR clonal lineage, as suggested for the European clone of *S*. 1,4,[5],12:i:- ST34 [16]. Moreover, these *S*. 1,4,[5],12:i:- copper tolerant strains might also have the ability to escape the metal-mediated antimicrobial response of human macrophages [70]. In the context of antibiotic reducing/replacing, the extensive use of heavy metals for animal growth promotion (copper and zinc) as well as mercury and arsenic compounds accumulation in the agriculture environment [17], might be potential factors contributing to the persistence and expansion of this emerging *S*. 1,4,[5],12:i:- ST3478 clonal lineage.

## 4. Conclusions

In summary, we report a low occurrence of *Salmonella* serotypes of Public Health significance in raw chicken carcasses in a poultry processing facility in Portugal, indicating the successful implementation of control practices in avian production. However, food safety authorities and Public Health laboratories should be aware of unusual non-H_2_S-producing *Salmonella* strains, currently circulating in diverse sources worldwide. This phenotype is especially worrisome since these strains may go undetected on the traditional medium because of their lack of black color, supporting the utility of chromogenic media and the combination of cultural- and molecular-based methods (as performed for STEC detection). Moreover, non-H_2_S-production, in combination with the ability of these strains to grow under diverse stresses (antibiotics, copper, acid pH, and peracetic acid), may be associated with their ongoing national and international spread. All these adaptive features also anticipates a future persistence and expansion of *S*. 1,4,[5],12:i:- ST3478 clonal lineage, due to an increased probability of selection throughout the food chain, thereby leading to a high risk of infection. This study alerts for the need of constant evaluation of biosafety measures to prevent the spread of new emerging pathogens in the poultry production to the final consumer and new challenges in the surveillance and control of *Salmonella* in the food chain.

## Figures and Tables

**Table 1:** Characterization of *Salmonella* isolates recovered from chicken meat samples.

Serotype (no. Isolates)/ST	No. Samples/Farm/Season	Antibiotic Resistance Phenotype/Genotype ^1^	Metal Tolerance Genes ^2^	MIC Copper Anaerobiosis (mM)	Minimum Growth pH ^3^	Minimum Survival pH	MIC Peracetic Acid (mg/L) ^3^		MBC Peracetic Acid (mg/L)	
							pH Not Adjusted	pH Adjusted to 4.5	pH Not Adjusted	pH Adjusted to 4.5
1,4,[5],12:i:- (*n* = 6)/ST3478	1 sample/farm A/spring	ASSu[T]/ *bla*_TEM_, *strA-strB*, *sul2*, [*tet(B)*]	*pcoD*, *silA*, *arsB*, [*merA*]	32	4.00	3.50–4.00	60–70	40	70–90	50
Enteritidis (*n* = 3)/ST11	1 sample/farm B/summer	-	-	4	4.00–4.50	4.00	60–70	20–30	90	40

Abbreviations: A, Ampicillin; S, Streptomycin; Su, Sulfamethoxazole; T, Tetracycline; MBC, Minimum Bactericidal Concentration; MIC, Minimum Inhibitory Concentration; ST, Sequence Type. ^1^ The square brackets represent a variable presence of antibiotic resistance phenotypes and genotypes among isolates. ^2^ The square brackets represent a variable presence of metal tolerance genes among isolates. ^3^ *Salmonella* Typhimurium LT2 (minimal growth/survival pH = 4.00, MIC_PAA_ = 60 mg/L and MBC_PAA_ = 80 mg/L) and *Enterococcus faecalis* ATCC 29212 (minimal growth pH = 4.50, minimal survival pH = 4.00, MIC_PAA_ = 120 mg/L and MBC_PAA_ = 130 mg/L) were used as controls in acid pH and PAA assays.

**Table 2:** Epidemiological background of atypical non-H_2_S-producing *Salmonella* strains with *pshA* gene mutations that resulted in a non-functional protein.

Serotype (ST - no. Isolates)	Source, Country (Year)	Type of *pshA* Gene Mutation ^1^ (Nucleotide, Protein)	Reference (Original Database; Accession no.) ^2^
1,4,[5],12:i:- (3478 - *n* = 1)	Poultry meat, Portugal (2018)	Nonsense (1669TC > T, Q557X)	This study
1,4,[5],12:i:- (3478 - *n* = 1)	Human-clinical (small tomatoes, outbreak), Sweden (2019)	Nonsense (1669C > T, Q557X)	[57] (ENA; ERR3577233)
Aberdeen (426 - *n* = 7)	Human-clinical, vegetables, surface water, China (2006–2013)	Nonsense (208C > T, Q70X)	[63] (GenBank; KU143714–KU143732)
Choleraesuis (68 - *n* = 19)	Human-clinical, China (2010–2011)	Frameshift (760delG)	[64] (GenBank; KP184398–184416, 18419–18420)
Choleraesuis 4(68 - *n* = 6)	Human, China (2010, 2012, 2013)	Frameshift (760delG)	[65] (GenBank; KY211936 to KY211941)
Havana (1621 - *n* = 3)	Broiler chickens, Turkey (2016, 2018)	Nonsense (1914C > A, Y638X)	[66] (GenBank; MK548410 to MK548412)
Infantis (32 - *n* = 1)	Poultry meat, Japan (2010)	Nonsense (358G > A, Q120X)	[58] (DDBJ; DRA000592)
Paratyphi A (85, 129 - *n* = 6)	Human, China (2010–2013)	Frameshift (1087delA)	[65] (GenBank; KY211950 to KY211955)
Senftenberg (185 - *n* = 1, 210 - *n* = 1, 217 - *n* = 1, 1751 - *n* = 14)	Seafood product and human-clinical, China (2005–2011)	Nonsense (1621C > T, Q541X)	[60] (GenBank; KF977150 to KF977170)
Typhimurium (328 - *n* = 3)	Poultry meat, Japan (2010)	Nonsense (1440C > A, C480X)	[58] (DDBJ; DRA000592)
Typhimurium (1544 - *n* = 1)	Human, China (2010)	Frameshift (1087delA)	[65] (GenBank; KY211942)

^1^ The nucleotide and protein sequences of *phsA* from non-H_2_S-producing *Salmonella* strains were compared with the one of H_2_S-producing *S*. Typhimurium LT-2 (GenBank accession no. L32188.1). IUAPC nucleotide (A, adenine; C, cytosine; G, guanine; T, thymine) and amino acid (C, cysteine; Q, glutamine; Y, tyrosine; X, stop codon) one letter code. ^2^ DDBJ, DNA Data Bank of Japan (https://www.ddbj.nig.ac.jp); ENA, European Nucleotide Archive (https://www.ebi.ac.uk/ena); GenBank at NCBI, National Center for Biotechnology Information (https://www.ncbi.nlm.nih.gov/); SRA, Sequence Read Archive (https://www.ncbi.nlm.nih.gov/sra).

## Supplementary Materials

The following are available online at https://www.mdpi.com/2076-0817/9/9/701/s1. Supplementary information includes four tables. Table S1. List of primers and conditions used in the standard PCR assays. Table S2. Metadata available from Enterobase as well as acquired genes and chromosomal mutations conferring antibiotic resistance and metal tolerance in the 16 analyzed ST3478 *Salmonella* strains. Table S3. Results of cgMLST and hierarchical clustering (HC) of the *Salmonella* ST3478 strains obtained through Enterobase pipeline. Table S4. Results of mapping SNPs of *Salmonella* ST3478 genomes obtained with the CSI Phylogeny-based pipeline. References [71,72,73,74,75,76] are cited in the supplementary materials.

## References

[1] Antunes P., Mourão J., Campos J., Peixe L. (2016). Salmonellosis: The role of poultry meat. Clin. Microbiol. Infect..

[2] European Food Safety Authority (EFSA), European Centre for Disease Prevention and Control (ECDC) (2019). The European Union One Health 2018 Zoonoses Report. EFSA J..

[3] The European Parliament, Council of the European Union (2003). Regulation (EC) No 2160/2003 of the European Parliament and of the Council of 17 November 2003 on the Control of Salmonella and Other Specified Food-Borne Zoonotic Agents.

[4] The European Commission (2011). Commission Regulation (EU) No 1086/2011 of 27 October 2011 Amending Annex II to Regulation (EC) No 2160/2003 of the European Parliament and of the Council and Annex I to Commission Regulation (EC) No 2073/2005 as Regards Salmonella in Fresh Poultry Meat.

[5] The European Commission (2012). Commission Regulation (EU) No 200/2012 of 8 March 2012 Concerning a Union Target for the Reduction of Salmonella Enteritidis and Salmonella Typhimurium in Flocks of Broilers, as Provided for in Regulation (EC) No 2160/2003 of the European Parliament and of the Council.

[6] Lin D., Yan M., Lin S., Chen S. (2014). Increasing prevalence of hydrogen sulfide negative *Salmonella* in retail meats. Food Microbiol..

[7] Campos J., Mourão J., Silveira L., Saraiva M., Correia C.B., Maçãs A.P., Peixe L., Antunes P. (2018). Imported poultry meat as a source of extended-spectrum cephalosporin-resistant CMY-2-producing *Salmonella* Heidelberg and *Salmonella* Minnesota in the European Union, 2014–2015. Int. J. Antimicrob. Agents.

[8] European Food Safety Authority (EFSA) (2019). The European Union summary report on antimicrobial resistance in zoonotic and indicator bacteria from humans, animals and food in 2017. EFSA J..

[9] Koutsoumanis K., Allende A., Alvarez-Ordóñez A., Bolton D., Bover-Cid S., Chemaly M., Cesare A.D., Herman L., Hilbert F., Lindqvist R. (2019). *Salmonella* control in poultry flocks and its public health impact. EFSA J..

[10] European Medicines Agency (EMA) (2016). Updated Advice on the Use of Colistin Products in Animals within the European Union: Development of Resistance and Possible Impact on Human and Animal Health.

[11] (2017). European Medicines Agency (EMA); European Food Safety Authority (EFSA). Joint Scientific Opinion on measures to reduce the need to use antimicrobial agents in animal husbandry in the European Union, and the resulting impacts on food safety (RONAFA). EFSA J..

[12] International Organization for Standardization (ISO) (2017). ISO 6579-1:2017; Microbiology of the Food Chain—Horizontal Method for the Detection, Enumeration and Serotyping of Salmonella—Part 1: Detection of Salmonella spp..

[13] Pritchett L.C., Konkel M.E., Gay J.M., Besser T.E. (2000). Identification of DT104 and U302 phage types among *Salmonella* enterica serotype typhimurium isolates by PCR. J. Clin. Microbiol..

[14] Héritier C., Poirel L., Aubert D., Nordmann P. (2003). Genetic and Functional Analysis of the Chromosome-Encoded Carbapenem-Hydrolyzing Oxacillinase OXA-40 of *Acinetobacter baumannii*. AAC.

[15] Tennant S.M., Diallo S., Levy H., Livio S., Sow S.O., Tapia M., Fields P.I., Mikoleit M., Tamboura B., Kotloff K.L. (2010). Identification by PCR of Non-typhoidal *Salmonella enterica* Serovars Associated with Invasive Infections among Febrile Patients in Mali. PLoS Negl. Trop. Dis..

[16] Mourão J., Novais C., Machado J., Peixe L., Antunes P. (2015). Metal tolerance in emerging clinically relevant multidrug-resistant *Salmonella* enterica serotype 4,[5],12:i: – clones circulating in Europe. Int. J. Antimicrob. Agents.

[17] Mourão J., Rebelo A., Ribeiro S., Peixe L., Novais C., Antunes P. (2020). Tolerance to arsenic contaminant among multidrug-resistant and copper-tolerant *Salmonella* successful clones is associated with diverse operons and genetic contexts. Environ. Microbiol..

[18] Mourão J., Marçal S., Ramos P., Campos J., Machado J., Peixe L., Novais C., Antunes P. (2016). Tolerance to multiple metal stressors in emerging non-typhoidal MDR *Salmonella* serotypes: A relevant role for copper in anaerobic conditions. J. Antimicrob. Chemother..

[19] European Committee of Antimicrobial Susceptibility Testing (EUCAST) (2020). Breakpoint tables for interpretation of MICs and zone diameters. https://www.eucast.org/fileadmin/src/media/PDFs/EUCAST_files/Breakpoint_tables/v_10.0_Breakpoint_Tables.pdf.

[20] Clinical and Laboratory Standards Institute (CLSI) (2016). Performance Standards for Antimicrobial Susceptibility Testing.

[21] European Committee of Antimicrobial Susceptibility Testing (EUCAST) (2016). Recommendations for MIC determination of colistin (polymyxin E) As recommended by the joint CLSI-EUCAST Polymyxin Breakpoints Working Group. https://www.eucast.org/fileadmin/src/media/PDFs/EUCAST_files/General_documents/Recommendations_for_MIC_determination_of_colistin_March_2016.pdf.

[22] International Organization for Standardization (ISO) (2006). ISO 20776-1:2006: Clinical Laboratory Testing and in Vitro Diagnostic Test Systems—Susceptibility Testing of Infectious Agents and Evaluation of Performance of Antimicrobial Susceptibility Test Devices—Part 1: Reference Method for Testing the In Vitro Activity of Antimicrobial Agents against Rapidly Growing Aerobic Bacteria Involved in Infectious Diseases.

[23] Clinical and Laboratory Standards Institute (CLSI) (1999). Methods for Determining Bactericidal Activity of Antimicrobial Agents.

[24] El-Azizi M., Farag N., Khardori N. (2016). Efficacy of selected biocides in the decontamination of common nosocomial bacterial pathogens in biofilm and planktonic forms. Comp. Immunol. Microbiol. Infect. Dis..

[25] Dean J.A., Lange N.A. (1985). Lange’s Handbook of Chemistry.

[26] Bankevich A., Nurk S., Antipov D., Gurevich A.A., Dvorkin M., Kulikov A.S., Lesin V.M., Nikolenko S.I., Pham S., Prjibelski A.D. (2012). SPAdes: A New Genome Assembly Algorithm and Its Applications to Single-Cell Sequencing. J. Comput. Biol..

[27] Aziz R.K., Bartels D., Best A.A., DeJongh M., Disz T., Edwards R.A., Formsma K., Gerdes S., Glass E.M., Kubal M. (2008). The RAST server: Rapid annotations using subsystems technology. BMC Genom..

[28] Zankari E., Hasman H., Cosentino S., Vestergaard M., Rasmussen S., Lund O., Aarestrup F.M., Larsen M.V. (2012). Identification of acquired antimicrobial resistance genes. J. Antimicrob. Chemother..

[29] Alikhan N.-F., Zhou Z., Sergeant M.J., Achtman M. (2018). A genomic overview of the population structure of Salmonella. PLoS Genet..

[30] Yoshida C.E., Kruczkiewicz P., Laing C.R., Lingohr E.J., Gannon V.P.J., Nash J.H.E., Taboada E.N. (2016). The Salmonella In Silico Typing Resource (SISTR): An open web-accessible tool for rapidly typing and subtyping draft *Salmonella* genome assemblies. PLoS ONE.

[31] Zhou Z., Alikhan N.-F., Sergeant M.J., Luhmann N., Vaz C., Francisco A.P., Carriço J.A., Achtman M. (2018). GrapeTree: Visualization of core genomic relationships among 100,000 bacterial pathogens. Genome Res..

[32] Zhou Z., Alikhan N.-F., Mohamed K., Fan Y., Achtman M., Brown D., Chattaway M., Dallman T., Delahay R., Kornschober C. (2020). The EnteroBase user’s guide, with case studies on Salmonella transmissions, Yersinia pestis phylogeny, and Escherichia core genomic diversity. Genome Res..

[33] Kaas R.S., Leekitcharoenphon P., Aarestrup F.M., Lund O. (2014). Solving the problem of comparing whole bacterial genomes across different sequencing platforms. PLoS ONE.

[34] Letunic I., Bork P. (2016). Interactive tree of life (iTOL) v3: An online tool for the display and annotation of phylogenetic and other trees. Nucleic Acids Res..

[35] Lin L., Zheng Q., Lin J., Yuk H.-G., Guo L. (2020). Immuno- and nucleic acid-based current technique for *Salmonella* detection in food. Eur. Food Res. Technol..

[36] Bell R.L., Jarvis K.G., Ottesen A.R., McFarland M.A., Brown E.W. (2016). Recent and emerging innovations in *Salmonella* detection: A food and environmental perspective. Microb. Biotechnol..

[37] Silveira L., Pista Â., Machado J. (2018). Caracterização fenotípica de isolados de *Salmonella enterica* recebidos no INSA entre 2014 e 2017. Boletim Epidemiológico Observações.

[38] Lin J., Lee I.S., Frey J., Slonczewski J.L., Foster J.W. (1995). Comparative analysis of extreme acid survival in *Salmonella* typhimurium, *Shigella flexneri*, and *Escherichia coli*. J. Bacteriol..

[39] Bearson S., Bearson B., Foster J.W. (1997). Acid stress responses in enterobacteria. FEMS Microbiol. Lett..

[40] Koutsoumanis K.P., Kendall P.A., Sofos J.N. (2004). Modeling the boundaries of growth of *Salmonella* Typhimurium in broth as a function of temperature, water activity, and pH. J. Food Prot..

[41] Hu S., Yu Y., Zhou D., Li R., Xiao X., Wu H. (2018). Global transcriptomic acid tolerance response in *Salmonella* Enteritidis. LWT.

[42] Joerger R.D., Sartori C., Frye J.G., Turpin J.B., Schmidt C., McClelland M., Porwollik S. (2012). Gene expression analysis of *Salmonella enterica* Enteritidis Nal ^R^ and *Salmonella enterica* Kentucky 3795 exposed to hcl and acetic acid in rich medium. Foodborne Pathog. Dis..

[43] Lianou A., Nychas G.-J.E., Koutsoumanis K.P. (2017). Variability in the adaptive acid tolerance response phenotype of *Salmonella enterica* strains. Food Microbiol..

[44] Joerger R.D., Sartori C.A., Kniel K.E. (2009). Comparison of genetic and physiological properties of *Salmonella enterica* isolates from chickens reveals one major difference between Serovar Kentucky and Other Serovars: Response to acid. Foodborne Pathog. Dis..

[45] Dubois-Brissonnet F., Annous B. (2012). Adaptation of *Salmonella* to Antimicrobials in Food-Processing Environments. Salmonella—Distribution, Adaptation, Control Measures and Molecular Technologies.

[46] Alonso-Hernando A., Alonso-Calleja C., Capita R. (2010). Effects of exposure to poultry chemical decontaminants on the membrane fluidity of *Listeria monocytogenes* and *Salmonella enterica* strains. Int. J. Food Microbiol..

[47] Humayoun S.B., Hiott L.M., Gupta S.K., Barrett J.B., Woodley T.A., Johnston J.J., Jackson C.R., Frye J.G. (2018). An assay for determining the susceptibility of *Salmonella* isolates to commercial and household biocides. PLoS ONE.

[48] Micciche A.C., Feye K.M., Rubinelli P.M., Lee J.A., Knueven C.J., Ricke S.C. (2019). Comparison of acid sanitizers on *Salmonella* Typhimurium inoculated commercial poultry processing reuse water. Front. Sustain. Food Syst..

[49] Jolivet-Gougeon A., Sauvager F., Bonnaure-Mallet M., Colwell R.R., Cormier M. (2006). Virulence of viable but nonculturable *S*. Typhimurium LT2 after peracetic acid treatment. Int. J. Food Microbiol..

[50] Bauermeister L.J., Bowers J.W.J., Townsend J.C., McKee S.R. (2008). The microbial and quality properties of poultry carcasses treated with peracetic acid as an Antimicrobial Treatment. Poult. Sci..

[51] Mathew E.N., Muyyarikkandy M.S., Bedell C., Amalaradjou M.A. (2018). Efficacy of Chlorine, Chlorine Dioxide, and Peroxyacetic Acid in Reducing *Salmonella* Contamination in wash water and on mangoes under simulated mango packinghouse washing operations. Front. Sustain. Food Syst..

[52] Lelieveld H., Holah J., Napper D. (2014). Hygiene in Food Processing.

[53] European Chemicals Agency (ECHA), Biocidal Products Committee (BPC) (2015). Opinion on the application for approval of the active substance: Paracetic Acid—Product type: 4. https://echa.europa.eu/documents/10162/24380804/7864_AS-APP_Peracetic+acid_PT4_Final+opinion.pdf/f077147e-e152-fc24-4f46-0b971a0fb25a.

[54] European Chemicals Agency (ECHA), Biocidal Products Committee (BPC) (2015). Regulation (EU) No 528/2012 concerning the making available on the market and use of biocidal products – Evaluation of active substances, Assessment Report of Peracetic Acid (Product Type 1-6). https://echa.europa.eu/documents/10162/24380810/8376_1340-04_Assessment_Report.pdf.

[55] Cadena M., Kelman T., Marco M.L., Pitesky M. (2019). Understanding Antimicrobial Resistance (AMR) Profiles of *Salmonella* Biofilm and Planktonic Bacteria Challenged with Disinfectants Commonly Used During Poultry Processing. Foods.

[56] Jourdan-da Silva N., Fabre L., Robinson E., Fournet N., Nisavanh A., Bruyand M., Mailles A., Serre E., Ravel M., Guibert V. (2018). Ongoing nationwide outbreak of *Salmonella* Agona associated with internationally distributed infant milk products, France, December 2017. Eurosurveillance.

[57] Colombe S., Jernberg C., Löf E., Angervall A.L., Mellström-Dahlgren H., Dotevall L., Bengnér M., Hall I., Sundqvist L., Kühlmann-Berenzon S. (2019). Outbreak of unusual H2S-negative monophasic *Salmonella* Typhimurium strain likely associated with small tomatoes, Sweden, August to October 2019. Eurosurveillance.

[58] Sakano C., Kuroda M., Sekizuka T., Ishioka T., Morita Y., Ryo A., Tsukagoshi H., Kawai Y., Inoue N., Takada H. (2013). Genetic Analysis of Non-Hydrogen Sulfide-Producing *Salmonella enterica* Serovar Typhimurium and *S*. enterica Serovar Infantis Isolates in Japan. J. Clin. Microbiol..

[59] Albert M.J., Al Obaid K., Alfouzan W., Sheikh A.R., Udo E., Izumiya H., Bulach D.M., Seemann T. (2014). Isolation of *Salmonella enterica* Serovar Kentucky Strain ST 198 and Its H2S-Negative Variant from a Patient: Implications for Diagnosis. J. Clin. Microbiol..

[60] Yi S., Xie J., Liu N., Li P., Xu X., Li H., Sun J., Wang J., Liang B., Yang C. (2014). Emergence and prevalence of Non-H2S-Producing *Salmonella enterica* serovar senftenberg isolates belonging to novel sequence type 1751 in China. J. Clin. Microbiol..

[61] Heinzinger N.K., Fujimoto S.Y., Clark M.A., Moreno M.S., Barrett E.L. (1995). Sequence analysis of the phs operon in *Salmonella* typhimurium and the contribution of thiosulfate reduction to anaerobic energy metabolism. J. Bacteriol..

[62] Rohmer L., Hocquet D., Miller S.I. (2011). Are pathogenic bacteria just looking for food? Metabolism and microbial pathogenesis. Trends Microbiol..

[63] Wu F., Xu X., Xie J., Yi S., Wang J., Yang X., Yang C., Liang B., Ma Q., Li H. (2016). Molecular characterization of *Salmonella enterica* Serovar Aberdeen Negative for H2S production in China. PLoS ONE.

[64] Xie J., Yi S., Zhu J., Li P., Liang B., Li H., Yang X., Wang L., Hao R., Jia L. (2015). Antimicrobial resistance and molecular investigation of H2S-Negative *Salmonella enterica* subsp. *enterica* serovar Choleraesuis Isolates in China. PLoS ONE.

[65] Xie J., Wu F., Xu X., Yang X., Zhao R., Ma Q., Li P., Wang L., Hao R., Jia L. (2018). Antibiotic resistance and molecular characterization of the hydrogen sulfide-negative phenotype among diverse *Salmonella* serovars in China. BMC Infect. Dis.

[66] Müştak İ.B., Müştak H.K., Sarıçam S. (2020). Molecular characterisation of hydrogen sulfide negative *Salmonella enterica* serovar Havana. Antonie Van Leeuwenhoek.

[67] Pearce M.E., Alikhan N.-F., Dallman T.J., Zhou Z., Grant K., Maiden M.C.J. (2018). Comparative analysis of core genome MLST and SNP typing within a European *Salmonella* serovar Enteritidis outbreak. Int. J. Food Microbiol..

[68] Alba P., Leekitcharoenphon P., Carfora V., Amoruso R., Cordaro G., Di Matteo P., Ianzano A., Iurescia M., Diaconu E.L., Study Group E.-E.-A.N. (2020). Molecular epidemiology of *Salmonella* Infantis in Europe: Insights into the success of the bacterial host and its parasitic pESI-like megaplasmid. Microb. Genom..

[69] Mastrorilli E., Pietrucci D., Barco L., Ammendola S., Petrin S., Longo A., Mantovani C., Battistoni A., Ricci A., Desideri A. (2018). A comparative genomic analysis provides novel insights into the ecological success of the monophasic *Salmonella* serovar 4,[5],12:i:. Front. Microbiol..

[70] Hao X., Lüthje F.L., Qin Y., McDevitt S.F., Lutay N., Hobman J.L., Asiani K., Soncini F.C., German N., Zhang S. (2015). Survival in amoeba—A major selection pressure on the presence of bacterial copper and zinc resistance determinants? Identification of a “copper pathogenicity island.”. Appl. Microbiol. Biotechnol..

[71] Agron P.G., Walker R.L., Kinde H., Sawyer S.J., Hayes D.C., Wollard J., Andersen G.L. (2001). Identification by subtractive hybridization of sequences specific for Salmonella enterica Serovar Enteritidis. Appl Environ Microbiol..

[72] Rasheed J.K., Jay C., Metchock B., Berkowitz F., Weigel L., Crellin J., Steward C., Hill B., Medeiros A.A., Tenover F.C. (1997). Evolution of extended-spectrum beta-lactam resistance (SHV-8) in a strain of Escherichia coli during multiple episodes of bacteremia. Antimicrob. Agents Chemother..

[73] Guerra B., Junker E., Miko A., Helmuth R., Mendoza M.C. (2004). Characterization and localization of drug resistance determinants in multidrug-resistant, integron-carrying Salmonella enterica serotype Typhimurium strains. Microb Drug Resist..

[74] Kerrn M.B., Klemmensen T., Frimodt-Moller N., Espersen F. (2002). Susceptibility of Danish Escherichia strains isolated from urinary tract infections and bacteraemia, and distribution of sul genes conferring sulfonamide resistance. J. Antimicrob. Chemother..

[75] Perreten V., Boerlin P. (2003). A new sulfonamide resistance gene (sul3) in Escherichia coli is widespread in the pig population of Switzerland. Antimicrob. Agents Chemother..

[76] Liebert C.A., Wireman J., Smith T., Summers A.O. (1997). Phylogeny of mercury resistance (mer) operons of Gram-negative bacteria isolated from the fecal flora of primates. Appl. Environ. Microbiol..

